# The Treatment of Puerperal Infection

**Published:** 1898-02

**Authors:** 


### THE TREATMENT OF PUERPERAL INFECTION.

The following is an abstract of a paper by F. A. Lockhart, M.B., and C.M., Edin., Demonstrator of Gynecology, McGill University; Assistant Gynecologist to the Montreal General Hospital; Gynecologist to the Protestant Hospital for the Insane, Verdun, read before the Franklin County (Vermont) Medical Society, and published in the *Montreal Medical Journal* for August:

"The number of the methods of treating this malady shows only too clearly that no specific has yet been discovered, not even the much talked-of antistreptococcus serum.

"One of the most important factors in the question is the early diagnosis of the case. To begin with, in all cases where you have the onset of the symptoms of puerperal septicæmia appearing, the parts should be carefully examined and any lacerations thoroughly cleansed and repaired to prevent further absorption from taking place through them. The interior of the uterus should likewise be carefully examined for the presence of retained products of conception, and, if found, they should be removed. The safest instrument with which to effect the removal is the finger, or, if this fails, the dull wire curette. A sharp curette may be used, but not unless the operator has had a great deal of experience with this instrument, as, unless one is very careful, it is liable to remove uterine substance.

"At this point, I hope that I may be permitted to make a short digression from actual treatment in order to urge the importance of not placing too much reliance upon the temperature in diagnosing septic infection, no matter whether it be puerperal or not. The pulse will be found to be a much safer guide, as, while you almost never see a case of sepsis without a quickened pulse, you will not rarely run across cases in which there is almost no noticeable rise in temperature, I myself having seen several cases in which the temperature did not rise over 99-5 degrees F. Where you have a rapid pulse, headache, foul tongue and dry, hot skin in a puerperal woman, look out for septic infection, no matter what the temperature indicates.

"In the majority of cases where the patient develops a slight chill with rapid pulse, and possibly some rise in tempertture on the fourth or fifth day after labor, an intra-uterine douche of sublimate (1-3000) will usually rapidly check the process. If the first one does not do so, it is well to repeat it in five or six hours, when thoroughly swabbing out the uterus with either pure carbolic acid or else iodized phenol in addition will often have marvelously beneficial results.

"If the case is more severe, especially if the cervix and vagina are covered with false membrane, thorough curettage of the uterus and also even of the vagina is indicated and may require to be repeated, as it is exceedingly difficult to get the curette up to the orifices of the Fallopian tubes. For this reason it is well to use a very small curette. This operation ought to be followed by a copious douche (intra-uterine) of sublimate (1-3000), which should be succeeded by one of boiled water, after which the cavity should be carefully dried and then swabbed out with either pure carbolic acid or iodized phenol.

"A word now in regard to sublimate douching. Many are afraid to use it, in case they cause sublimate poisoning. While not denying that this does occasionally occur, especially in blondes, the sublimate douche, when properly given, seldom is followed by any ill results.

"Personally, when the intra-uterine douche is indicated, sublimate is what I always employ in a strength of 1-3000 both in public and private, and I have never yet seen any harmful effect follow; but great care is always taken to have a free exit for the fluid from the uterine cavity, and the last drops are expelled by expression.

"In the City of London Lying-in Hospital, Clement Godson uses sublimate entirely, using 1-1000 for the hands and 1-2000 for vaginal irrigation. He gives a 1-2000 vaginal douche at 115 degrees F., immediately after delivery of the placenta, and repeats it three times, with an interval of twelve hours between each, after which he replaces the sublimate with iodine. This method has been employed in over 4,500 deliveries without any sign of mercurial poisoning. Another British writer, Sharp, says that 'in a really serious septic case, corrosive sublimate is the only reliable antiseptic, and should be used fearlessly in a strength of 1-2000.' This, however, I think is too strong a solution, as such a powerful one would destroy the tissues, and so furnish pabulum for the germs.

"In addition to local treatment, the patient's general condition will require attention. Her strength must be maintained by nourishing but easily digested food, such as milk, eggs, soups, juice expressed from beef, which has just been warmed through, etc. Stimulants are usually necessary, good brandy, whisky or port being the most serviceable of the alcoholic ones, and strychnine of the truly medicinal ones, giving gr. one-forty to one-thirty every four, six or eight hours, as the urgency of the case requires. The spirits may be beaten up with egg and milk once or twice daily, if the patient can stand these latter, in addition to taking them in water at whatever intervals are indicated.

"The bowels and other emunctories should be kept active, and hot applications to the abdomen, especially turpentine stupes, are of great service in relieving the pain. For this latter purpose, Battley's solution is very useful, if acting somewhat as a stimulant in addition to its sedative properties.

"It is not advisable to give antipyretics continuously, as they simply obscure the symptoms, but eight or ten grains of quinine may be given now and then if the temperature keeps up too high.

"Serum theraphy in the treatment of puerperal septicæmia has been receiving considerable attention of late and numerous cures by it have been reported, but Marmorek's antistreptococcus serum is still on its trial, and, I fear, will not turn out to be such a success as was at first hoped. In nearly all of the successful cases in which it has been used, the general and local treatment above recorded has been employed, which utterly negetives the results as far as testing the value of the serum is concerned. Certainly, a few cases have been reported where its administration has been followed by rapid amelioration of the symptoms, although the older treatment had been thoroughly employed with no effect, so that there is no doubt but that it is sometimes of benefit. It must be remembered, however, that the majority of mild cases, if taken in hand early, get well under intra-uterine douching, and that many of the severer ones can be cured by curetting combined with the application of caustics and douching, while other cases, apparently no more severe, will result fatally, no matter what we do. The dose of the serum varies, rapid improvement having followed the injection of 3 c. cm., while 20 c. cm. have been used in other cases with absolutely no result. The dose ordinarily employed seems to be 10 c. cm., injected under the skin of the abdomen, and repeated every six or eight hours if required. When successful, an almost immediate fall of temperature and pulse-rate follow. The only complication which has been recorded is the occurrence occasionally of a bullous eruption near the seat of injection. Whether one uses the serum or not, it is absolutely necessary that he should not neglect to employ the older treatment in addition.

"From an analysis of the cases fully reported where it has been employed, the serum appears to give excellent results where cultures prove the presence of streptococci alone, but, where the infection is mixed it is of but little use.

"As regards operations for relief of the condition, I may say that curetting is the only one to be recommended, although, of course, abscesses which develop should be opened. When a case is sufficiently severe to indicate hysterectomy, it is too late to operate, and, as so many cases get well after less heroic methods of treatment, I don't think that we are justified in removing the uterus, which in itself is a grave undertaking. If, however, you find that the uterus is greatly lacerated, inviting the absorption of infection, as it were, the question of hysterectomy may be considered."—*Medical Review of Reviews.*

